# Endospore forming bacteria may be associated with maintenance of surgically-induced remission in Crohn’s disease

**DOI:** 10.1038/s41598-018-28071-z

**Published:** 2018-06-27

**Authors:** Michael R. Laffin, Troy Perry, Heekuk Park, Patrick Gillevet, Masoumeh Sikaroodi, Gilaad G. Kaplan, Richard N. Fedorak, Karen Kroeker, Levinus A. Dieleman, Bryan Dicken, Karen L. Madsen

**Affiliations:** 1 Department of Surgery, University of Alberta, Edmonton, Alberta Canada; 2 Department of Medicine, University of Alberta, Edmonton, Alberta Canada; 3 Microbiome Analysis Center, George Mason University, Manassas, Virginia USA; 4 Department of Medicine and Community Health Sciences, University of Calgary, Calgary, Alberta Canada

## Abstract

Crohn’s disease (CD) patients who undergo ileocolonic resection (ICR) typically have disease recurrence at the anastomosis which has been linked with a gut dysbiosis. The aims of this study were to define the mucosa-associated microbiota at the time of ICR and to determine if microbial community structure at the time of surgery was predictive of future disease relapse. Ileal biopsies were obtained at surgery and after 6 months from CD subjects undergoing ICR. Composition and function of mucosal-associated microbiota was assessed by 16S rRNA sequencing and PICRUSt analysis. Endoscopic recurrence was assessed using the Rutgeerts score. Analysis of mucosal biopsies taken at the time of surgery showed that decreased Clostridiales together with increased Enterobacteriales predicted disease recurrence. An increase in the endospore-forming Lachnospiraceae from surgery to 6 months post-ICR was associated with remission. A ratio of 3:1 between anaerobic endospore-forming bacterial families and aerobic families within the Firmicutes phylum was predictive of maintenance of remission. Gut recolonization following ICR is facilitated by microbes which are capable of either aerobic respiration or endospore formation. The relative proportions of these species at the time of surgery may be predictive of subsequent microbial community restoration and disease recurrence.

## Introduction

Crohn’s disease (CD) involves transmural inflammation of the alimentary tract with ~50% of patients requiring an intestinal resection within 10 years of diagnosis^1–3^. The most common resection performed for CD is an ileocolic resection (ICR), which involves removal of diseased areas in the ileum and right colon with ileocolonic anastomosis^3^. Unfortunately, disease tends to recur in the neo-terminal ileum in up to 85% of patients within one year of their resection^4–7^. Rutgeerts *et al*. reported that proximal fecal diversion prevented post-operative recurrence suggesting that the fecal stream and its microbial contents play a central role in disease recurrence^4^; however, the microbial constituents leading to postoperative recurrence remain elusive.

Several studies have evaluated the microbiota at the time of surgery in attempts to identify bacterial signatures that predict remission or relapse^5–11^. Intestinal resection constitutes a large insult to the resident microbiota and re-colonization depends upon which commensal organisms are able to survive and reproduce following surgery. Survival of organisms under such stress would require either the ability for aerobic respiration or alternatively the ability to sporulate during the insult. In murine models of ICR, Firmicutes dominate post-operatively^12,13^. This is not surprising given the phenotypic diversity of this phylum with aerobes, facultative and strict anaerobes, and species capable of forming endospores. Therefore, the pre-operative community composition within the Firmicutes phylum may determine how recolonization proceeds following surgery thereby modulating the post-surgical inflammatory environment. This study involved a prospectively collected cohort of post-ICR CD subjects. The aims were to define the mucosa-associated microbiota at the time of ICR and at 6 months following ICR, and to determine if the bacterial community structure at the time of surgery was predictive of future disease relapse. Herein, we show that the balance at the time of surgery between groups of bacteria in the Firmicutes phyla capable of either aerobic respiration or endospore formation was predictive of future disease relapse.

## Methods

### Patient Cohort

The present study was approved by the Health Research ethics board at the University of Alberta (Pro00028147) and all methods performed in accordance within relevant guidelines and regulations set out by the ethics board. Forty-five subjects were recruited and provided informed consent by either ML or TP at the University of Alberta Hospital, Royal Alexandra Hospital, Grey Nuns Hospital, or Misericordia Hospital prior to their operation. Between June 2012 and June 2015, CD patients (n = 45) undergoing ICR were recruited. Exclusion criteria included antibiotics within one month of surgery, creation of a diversion ostomy, age less than 18 years old, inability to consent, and women who were pregnant or wishing to become pregnant. Microbial data for 24 subjects was produced for both time points, 14 only at surgery, and 8 only at colonoscopy. Missing data is due to inadequate DNA extraction (n = 12), change in residence making sample collection not possible (n = 2), and refusal of additional biopsies at the time of ileocolonscopy (n = 2). 6 months post-operatively endoscopic remission was assessed using the validated Rutgeerts score with recurrence being defined as ≥2 (Supplementary Table 1)^14^. On the day of surgery and at post-surgical ileocolonoscopy, the subject’s medical record was reviewed. The review included demographic information, disease behavior, smoking history, past surgical history, and recent CD-related medication usage. All subjects received intravenous antibiotics immediately pre-operatively (cefazolin and metronidazole or clindamycin and gentamycin in cases of β-lactam allergy). Subjects were phenotyped based on the Montreal Classification^15^.

### Sample collection

Mucosal biopsies were obtained at the time of resection. Biopsies were taken from the ileum of the surgical specimen in macroscopically normal tissue in the operating room immediately resection. At ileo-colonoscopy biopsies from non-ulcerated tissue in the neo-terminal ileum were obtained from within 5 cm of the anastomosis. All samples were taken using biopsy forceps sterilized prior to each subject. No washing procedures were preformed prior to samples being snap frozen in liquid nitrogen and stored at −80 °C.

### Assessment of tissue inflammation

Snap frozen biopsies were homogenized in PBS containing 0.05% Tween 20. Homogenates were centrifuged at 10 000 rpm for 10 minutes and cytokines measured in the supernatant (IL-2, IL-6, IL-8, and TNFα) using the Meso Scale discovery platform (Meso Scale Diagnostics, Gaithersburg, MD, USA).

### Microbial Composition

The FastDNA Spin Kit (MP Biomedicals, OH USA) was used to extract DNA as per the manufacturer’s protocol. This protocol involves a mechanical and chemical DNA extraction. DNA was quantified using PICO green assay. The 16s ribosomal RNA gene was sequenced using an Ion Torrent sequencer with 27F (5′-AGA GTT TGA TCC TGG CTC AG-3′) and 355R (5′-GCT GCC TCC CGT AGG AGT-3′) universal primers targeting the V1-V2 region^16^. Amplicons were purified using Agilent magnetic beads. Sequencing reads greater than 250 basepairs were selected to obtain high quality reads. After trimming for quality, a total of 1 320 578 reads were obtained from 70 ileal samples (n = 38 pre-operative samples and n = 32 post-operative samples). Sequence reads ranged from 617 to 56 192 with a median value of 18 772. Sequences were subsequently analyzed using RDP11 and QIIME pipelines using UCLUST to assign sequences, ChimeraSlayer was used for chimera detection and removal. Alpha diversity was analyzed on the OTU table rarefied to 617 reads using the Shannon diversity index. PICRUSt software was used to predict metagenomic functional content and categorized as level 1 to 3 into KEGG pathways^17^.

### Data analysis

Statistical analysis was performed using STATA, Galaxy, and QIIME software. Sample size was calculated using the work of Sokol *et al*.^9^ specifically related to changes in the proportion of the Firmicutes phyla. Therefore, the calculation was based on a relative Firmicutes amount of 20% in non-recurrent subjects, 15% in recurrent subjects, with a standard deviation of 8, requiring 41 subjects to produce a power of 0.8 and α of 0.05. Demographic and cytokine data was assessed using Student’s t-test for continuous variables after ensuring normality assumptions were met using the Shapiro-Wilk test, and Fisher’s exact test for categorical variables. Adjustment was performed using a multivariable logistic regression including factors that achieved a p-value < 0.1 on univariate analysis. Microbial comparisons were assessed using Kruskal-Wallis one-way test of variance on taxa present in greater than 75% of all samples. Student’s paired t-test was used on sets of data complete for both time points. False discovery rate (FDR) was corrected using the Benjamini-Hochberg procedure. Results where p ≤ 0.05 following FDR-adjustment will be referred to as “significant following FDR-adjustment”, whereas those results with a p-value ≤ 0.05 and an FDR-adjusted p-value > 0.05 will be referred to as “significant.” To identify factors with differentiating abundance in the different groups, the LDA (Linear Discriminant Analysis) Effect Size (LEfSe) algorithm was used with the online interface Galaxy (http://huttenhower.sph.harvard.edu/galaxy/root). The algorithm is designed to evaluate differential abundance along with potential biologic significance and involves the non-parametric factorial Kruskal-Wallis (KW) sum-rank test to identify populations with significant differential abundance between groups, a Wilcoxon rank-sum test to assess a biological significance and a Linear Discriminant Analysis that calculates the effect size of the differentially abundant feature. P values were set at 0.05 without FDR-adjustment and a linear discriminant analysis cut-off score of 3.0^18^. Partial least square analysis discriminant analysis (PLS-DA) was applied to cluster observations with similar microbial profiles. Data is presented as mean value ± the standard deviation (SD).

### Data availability

The microbial datasets generated and analysed during the current study are available from the corresponding author on reasonable request.

## Results

### Subject demographics

Subject demographics are detailed in Table 1. At 6 months, 30 subjects remained in endoscopic remission while 15 had recurrent disease. Clinical factors that had previously been associated with post-operative disease activity did not differ between groups; these included disease location, smoking status, and the presence of peri-anal disease. Proportions of remission and relapsing subjects using each class of CD medications, including antibiotic usage in the immediate post-operative period, was not significantly different at the time of surgery or at the time of follow-up colonoscopy. A detailed description of each subject’s clinical information is provided in Supplementary Table 2.

**Table 1:** Clinical and demographic characteristics of subjects by recurrence status.

	Recurrence (n = 15)	Remission (n = 30)	*p*
AGE (YEARS)	48.4	40.6	0.09
MALE (%)	20%	47%	0.11
PREVIOUS ICR (%)	60%	37%	0.21
AVERAGE TIME TO ENDOSCOPY (DAYS)	203	222	0.50
CURRENT SMOKER (%)	13%	10%	1.00
PERI-ANAL DISEASE (%)	13%	17%	1.00
DISEASE LOCATION			
*ILEAL (%)*	53%	73%	0.20
*COLONIC (%)*	0	0	
*ILEOCOLONIC (%)*	47%	27%	
DISEASE BEHAVIOUR			
*INFLAMMATORY (%)*	0%	7%	0.88
*STRICTURING (%)*	60%	60%	
*PENETRATING (%)*	40%	33%	
OPERATIVE INDICATIONS			
*OBSTRUCTION (%)*	93%	73%	0.36
*ENTERO-ENTERIC FISTULA (%)*	7%	17%	
*FAILURE OF MEDICAL THERAPRY (%)*	0%	10%	
AGE AT CD DIAGNOSIS			
*LESS THAN 16 YEARS*	7%	27%	0.35
*BETWEEN 16 AND 40 YEARS*	80%	60%	
*GREATER THAN 40 YEARS*	13%	13%	
PRE-OPERATIVE CD MEDICATIONS			
STEROID (%)	47%	27%	0.17
BIOLOGIC THERAPY (%)	53%	47%	0.76
5-ASA DRUG (%)	0%	20%	0.16
AZATHIOPRINE (%)	20%	47%	0.11
METHOTREXATE (%)	20%	7%	0.32
POST-OPERATIVE CD MEDICATIONS			
STEROID (%)	0%	7%	0.55
BIOLOGIC THERAPY (%)	47%	60%	0.55
5-ASA DRUG (%)	0%	17%	0.15
AZATHIOPRINE (%)	27%	40%	0.51
METHOTREXATE (%)	13%	3%	0.25
ANTIBIOTICS (%)	27%	47%	0.33

^*^p-value were calculated using Student’s t-test for continuous variables. Categorical variables were assessed using the Fisher’s exact test.

### The mucosal-associated microbial profile and its association with disease recurrence

**Figure 1:**
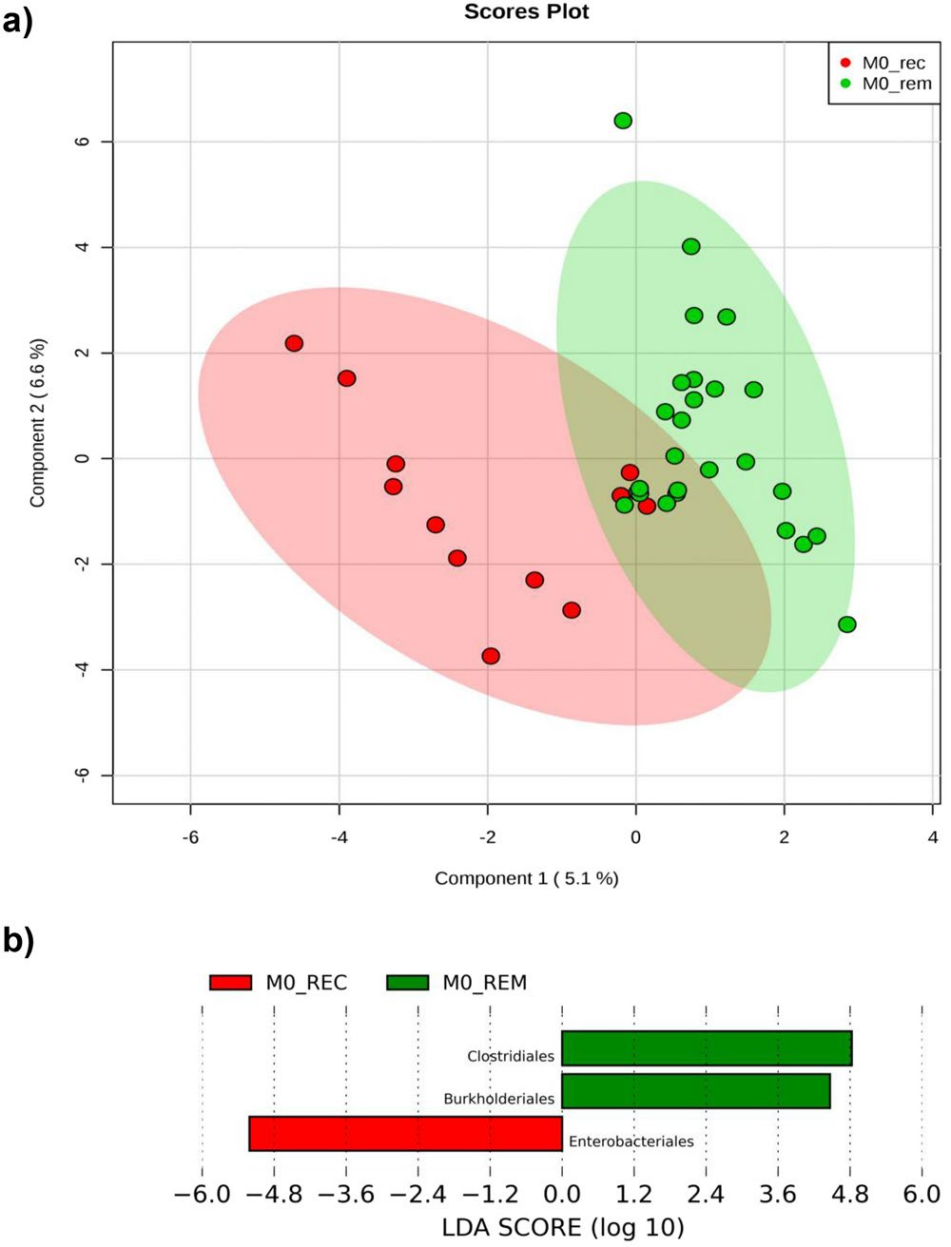
Partial least square analysis discriminant analysis (PLS-DA) of taxonomic data at baseline. (**a**) At the time of surgery, samples from subjects who remained in remission clustered separately (Remission:Green) from samples taken from subjects who had recurrence at month 6 (Recurrence:Red). (**b**) LEfSe analysis showed that Enterobacteriales in the recurrence cohort and Clostridiales and Burkholderiales in the remission cohort drove the separation.

Subjects with postoperative disease recurrence had significantly elevated Enterobacteriaceae (Remission: 16.9 ± 5.6%; Recurrence: 44.3 ± 10.4% p = 0.03, FDR-adjusted p = 0.11), and reduced Clostridiales (Remission: 19.5 ± 3.6%; Recurrence: 8.8 ± 2.1%, p = 0.02, FDR-adjusted p = 0.10) at the time of surgery compared with subjects which remained in remission. The two groups separated based upon their microbial composition (Fig. 1a) and LEfSe analysis showed Clostridiales and Burkholderiales to predict remission with Enterobacteriale associated with recurrence (Fig. 1b).

When analyzing changes that occurred within subjects between the time of surgery and six months post-ICR, differences were observed in subjects who remained in remission and those which relapsed (Table 2; Supplementary Figure 1). Recurrent subjects experienced a significant decrease in Actinobacteria (Recurrent subjects at surgery: 1.2% (1.0%); Recurrent subjects at follow-up: 0.3%(0.3%) p = 0.04, FDR-adjusted p = 0.16). In contrast, subjects who remained in remission demonstrated a significant increase in Lachnospiraceae, which was not equaled in the recurrent group (Remission subjects at surgery: 9.7% (7.2%); Remission subjects at follow-up: 23.8% (21.3%) p = 0.02, FDR-adjusted p = 0.08).

**Table 2:** Changes in bacterial populations from the time of surgery to follow up endoscopy in subjects with recurrence and remission.

Population	Surgical specimen in recurrent subjects (SD) n = 9	6-month post-ICR specimen in recurrent subjects (SD) n = 9	p-value	FDR-adjusted p-value	Surgical specimen in remission subjects (SD) n = 15	6-month post-ICR specimen in remission subjects (SD) n = 15	p-value	FDR-adjusted p-value
Phylum								
Proteobacteria	40.7% (37.6%)	19.4% (21.7%)	0.18	0.36	30.2% (31.7%)	27.0% (22.9%)	0.65	0.97
Bacteroidetes	36.3% (32.4%)	65.0% (30.4%)	0.08	0.24	44.8% (31.3%)	31.5% (32.0%)	0.10	0.30
Firmicutes	20.9% (19.7%)	15.0% (20.6%)	0.51	0.51	23.0% (22.5%)	39.8% (31.7%)	**0.04**	0.16
Actinobacteria	1.2% (1.0%)	0.3 (0.3%)	**0.04**	0.16	1.1% (1.8%)	1.1 (1.7%)	0.97	0.97
Class								
Bacteroidia	35.1% (33.7%)	64.5% (30.5%)	0.08	0.40	43.5% (31.8%)	29.9% (32.2%)	0.10	0.40
Clostridia	11.0% (7.8%)	13.9% (20.9%)	0.66	0.66	20.5% (19.9%)	36.5% (30.6%)	**0.04**	0.20
Gammaproteobacteria	37.0% (36.5%)	17.3% (21.1%)	0.20	0.58	25.4% (30.8%)	20.9% (19.3%)	0.48	0.56
Bacilli	7.2% (14.6%)	0.7% (1.2%)	0.23	0.58	2.0% (3.3%)	1.4% (2.2%)	0.56	0.56
Alphaproteobacteria	2.6% (2.4%)	1.6% (1.1%)	0.29	0.58	2.2% (6.2%)	4.0% (6.0%)	0.45	0.56
Order								
Bacteroidales	35.1% (33.7%)	64.5% (30.5%)	0.08	0.48	43.5% (31.8%)	29.9% (32.2%)	0.10	0.50
Clostridiales	11.0% (7.8%)	13.9% (20.9%)	0.66	0.66	20.5% (19.9%)	36.5% (30.6%)	0.04	0.24
Enterobacteriales	35.4% (27.9%)	16.6% (21.5%)	0.22	0.66	25.0% (30.8%)	17.7% (15.6%)	0.34	0.68
Rhizobiales	2.2% (2.5%)	1.6% (1.1%)	0.54	0.66	1.6% (4.1%)	3.9% (5.8%)	0.25	0.68
Lactobacillales	7.1% (14.4%)	0.7% (1.2%)	0.23	0.66	1.9% (3.2%)	1.3% (2.2%)	0.48	0.68
Burkholderiales	1.1% (1.6%)	0.5% (0.7%)	0.30	0.66	2.5% (3.7%)	2.0% (3.0%)	0.68	0.68
Family								
Bacteroidaceae	32.7% (31.1%)	61.4 (29.8%)	0.07	0.28	36.0% (28.9%)	28.2% (32.4%)	0.29	0.34
Enterobacteriaceae	35.4% (37.9%)	16.6% (21.5%)	0.22	0.44	25.0% (30.8%)	17.7% (15.6%)	0.34	0.34
Lachnospiraceae	5.7% (5.0%)	5.4% (6.5%)	0.85	0.85	9.7% (7.2%)	23.8% (21.3%)	**0.02**	0.08
Streptococcaceae	6.7% (13.7%)	0.3% (0.4%)	0.20	0.44	1.2% (1.7%)	0.6% (1.1%)	0.13	0.34

All values calculated using the Student paired t-test. FDR- corrected q-values derived using Benjamini-Hochberg procedure.

At the time of surgery α-diversity was similar in all subjects (Shannon index: Remission: 4.10 (0.87); Recurrence: 3.99 (0.83)) and did not change within individuals following surgery (Shannon index: Surgery: 4.17 (0.86); Month 6: 3.88 (0.86)). As expected, in all subjects’ surgery induced changes in mucosal-associated microbiota. At the family level, Lachnospiraceae was elevated at 6 months (Table 3) (p < 0.03, FDR-adjusted p = 0.12).

**Table 3:** Bacterial populations that differed significantly following surgery.

Population	Surgery (SD) n = 24	Month 6 (SD) n = 24	p	FDR-corrected q-value
**Phylum**				
Proteobacteria	34.1% (33.6%)	24.1% (22.3%)	0.17	0.68
Bacteroidetes	41.6% (31.3%)	44.0% (35.0%)	0.77	0.77
Firmicutes	22.2% (21.0%)	30.5% (30.2%)	0.33	0.77
Actinobacteria	1.1% (1.5%)	0.8% (1.4%)	0.42	0.77
**Class**				
Bacteroidia	40.3% (32.1%)	42.9% (35.3%)	0.76	0.76
Clostridia	16.9% (16.9%)	28.1% (29.1%)	**0.04**	0.20
Gammaproteobacteria	29.7% (33.0%)	19.6% (19.6%)	0.13	0.51
Bacilli	3.9% (9.3%)	1.1% (1.9%)	0.17	0.51
Alphaproteobacteria	2.4% (5.0%)	3.0% (4.8%)	0.63	0.76
**Order**				
Bacteroidales	40.3% (32.1%)	42.9% (35.3%)	0.76	0.66
Clostridiales	16.9% (16.9%)	28.1% (29.1%)	**0.04**	0.23
Enterobacteriales	28.9% (33.2%)	17.3% (17.6%)	0.11	0.55
Rhizobiales	1.8% (3.5%)	3.0% (4.7%)	0.35	0.76
Lactobacillales	4.7% (10.9%)	1.6% (3.1%)	0.28	0.76
Burkholderiales	2.0% (3.1%)	1.4% (2.5%)	0.48	0.76
**Family**				
Bacteroidaceae	34.8% (29.1%)	40.7% (34.9%)	0.44	0.44
Enterobacteriaceae	28.9% (33.2%)	17.3% (17.6%)	0.11	0.26
Lachnospiraceae	8.3% (6.7%)	16.9% (19.3%)	**0.03**	0.12
Streptococcaceae	3.3% (8.6%)	0.5% (0.9%)	0.13	0.26

^*^All values calculated using the Student paired t-test.

### Functional predictors of disease recurrence

**Figure 2:**
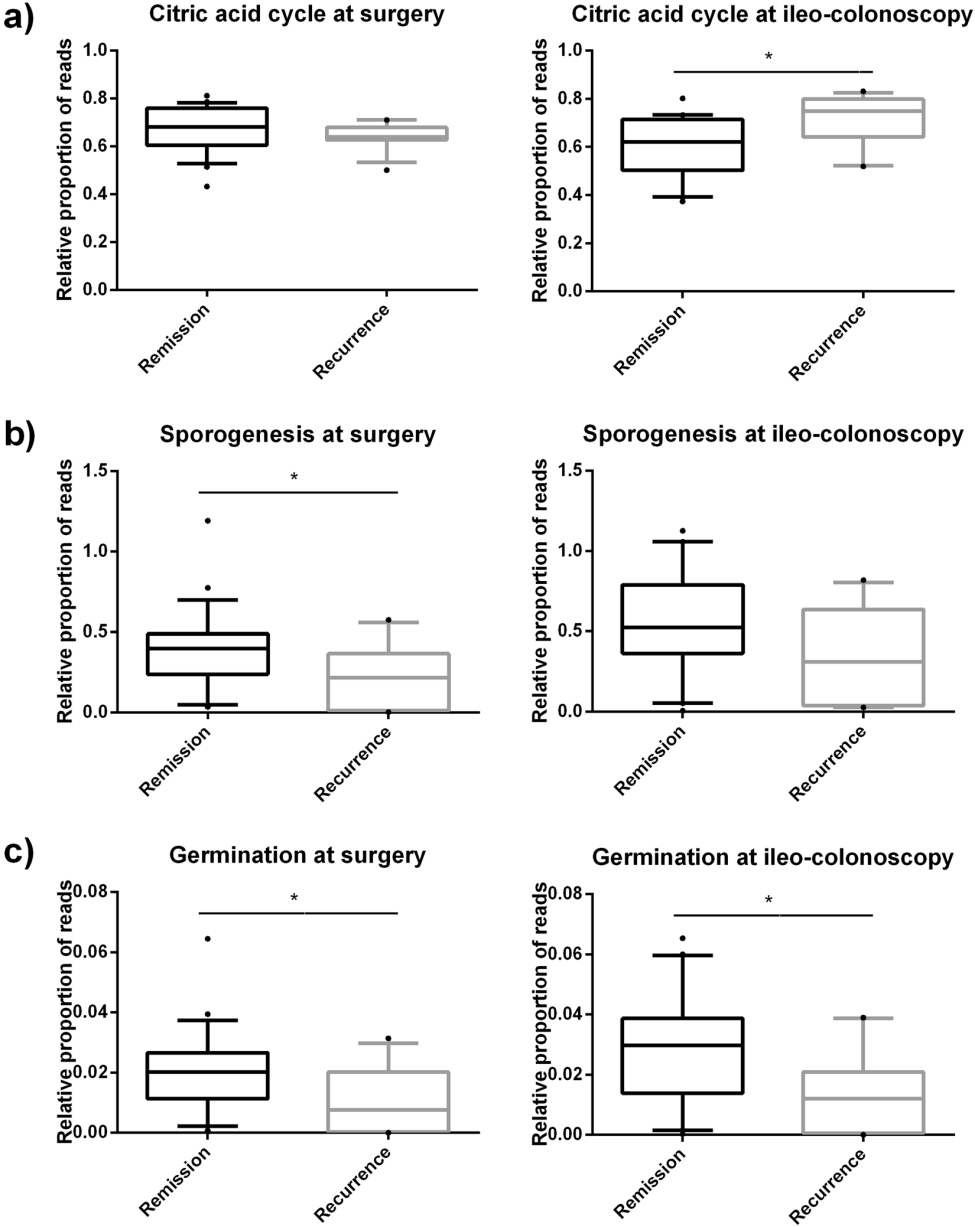
PICRUSt analysis of mucosal-associated microbiota showing relative abundance of specific functional pathways associated with bacterial sporogenesis, germination, and aerobic metabolism. At the time of surgery, samples taken from subjects who remained in remission had higher levels of sporogenesis (**b**) and germination (**c**) compared with samples taken from subjects who had disease recurrence. This remained higher at the time of ileo-colonoscopy. Subjects which had disease recurrence had increased proportion of genes related to aerobic respiration (citric acid cycle) (**a**). *p < 0.05.

**Figure 3:**
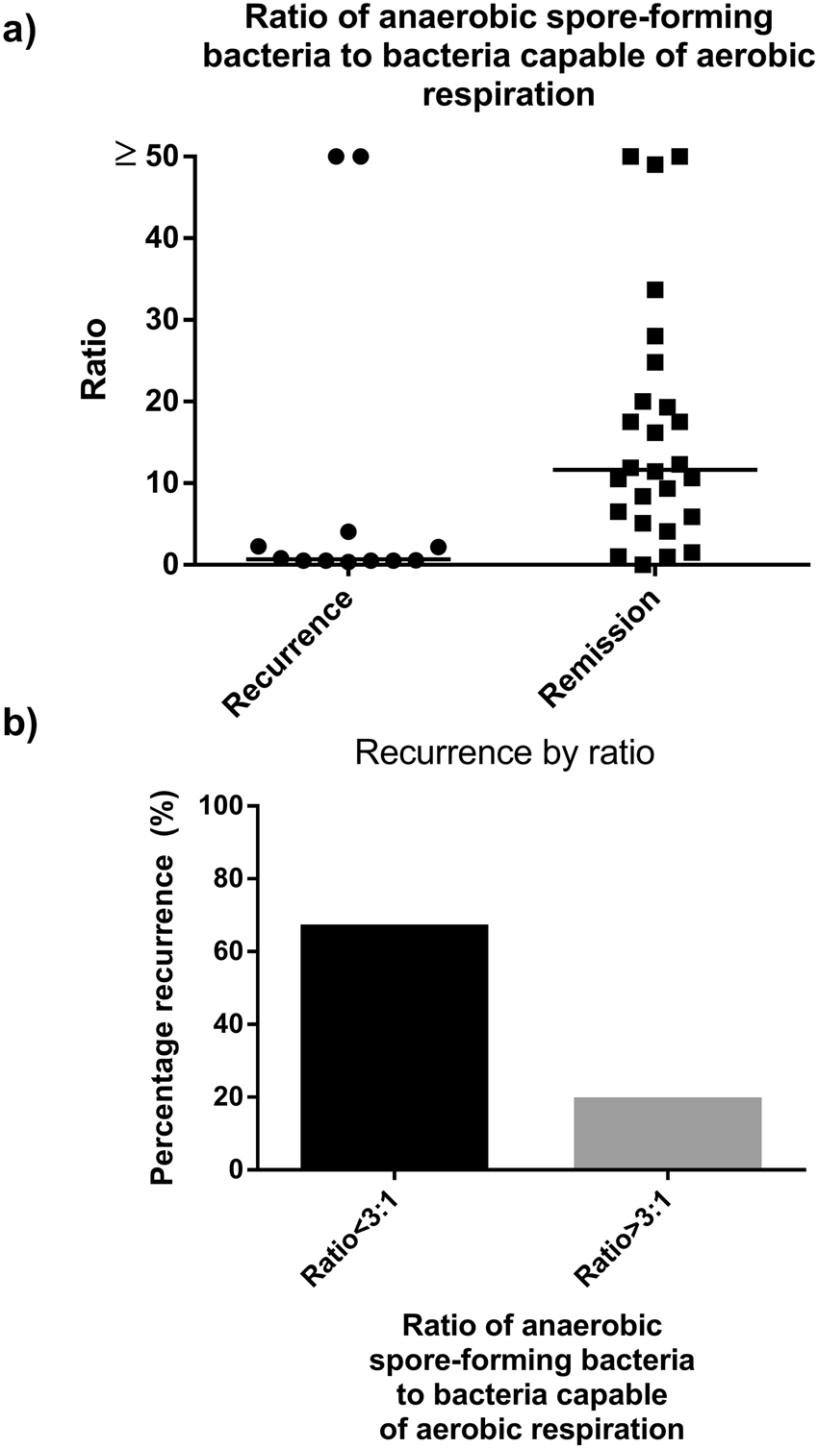
Ratio of obligatory anaerobic spore-forming bacteria to those capable of aerobic respiration within the Firmicutes phylum at the time of ICR. (**a**) Median ratio within the MAM of specimens collected at the time of ICR of obligatory anaerobic spore-forming families to families capable of aerobic respiration within the Firmicutes phylum (**b**) Rate of recurrence (%) in those with a ratio of >3:1 and <3:1 in terms of anaerobic endospore-forming bacteria and bacteria capable of aerobic respiration within the Firmicutes phylum. The rate of recurrence in those with a ratio of >3:1 was 19% and 67% in those with a ratio <3:1.

At the time of surgery, mucosal-associated microbiota in subjects which remained in remission possessed a higher proportion of reads associated with sporulation and germination (Fig. 2) while samples from subjects which had disease recurrence contained more reads associated with the citrate cycle (i.e. aerobic respiration). Based upon these findings, we examined the relationship within the Firmicutes phyla between families capable of aerobic respiration and families which contained anaerobic endospore-formers (Supplementary Table 3). Subsequent analysis revealed a relationship between recurrence and the ratio of anaerobic endospore-forming bacterial families to families capable of aerobic respiration present at the time of surgery. Overall, subjects which remained in remission had an increased ratio of anaerobic spore-forming bacteria to bacteria capable of aerobic respiration compared with subjects which had disease recurrence (Fig. 3a). A ratio greater than 3:1 between these two groups of bacteria was identified in 26 subjects, 21 of which remained in endoscopic remission. In the 12 subjects where the ratio was less than 3:1, 8 experienced recurrence. This represents an unadjusted odds ratio of 8.4 (95% CI 1.8–39.4 p < 0.01). After multivariable adjustment the odds ratio maintained significance (OR-9.2 95% CI 1.8–47.7 p < 0.01). In addition, a ratio of >3:1 at the time of surgery was associated with increased α-diversity at follow-up endoscopy (High ratio: 4.10 (0.79), Low ratio: 3.36 (0.69) p = 0.04).

### Recurrence and endospore content are independent of mucosal inflammation

To determine if differences in microbial composition and function were related to the degree of inflammation, levels of IL-2, IL-6, IL-8, and TNFα were measured in biopsies taken at the time of surgery. (Supplementary Figure 2). These cytokine profiles showed no correlation with subsequent disease recurrence or with any microbial signature, suggesting that this microbial signature was inflammation independent. We also examined TNFα levels as a function of whether the subject was on pre-operative anti-TNF therapy and there was a trend toward reduced mucosal levels of TNFα at the time of surgery in those on biologic therapy (Supplementary Figure 2).

## Discussion

In this study, we report an association between mucosal-associated microbiota at the time of surgery and post-operative recurrence of CD in a large cohort of post-operative CD patients. We also identify a potential impact of endospore-forming bacteria in the process of gut recolonization and post-operative CD recurrence. Finally, we propose a novel niche driven approach to the assessment of the microbiota at the time of intestinal resection.

The majority of colonic microbiota are made up of obligate anaerobic bacteria. Major physiologic stressors, such as intestinal surgery, can induce massive microbial ecological shifts^19^ and survival of local species depends on their ability to cope with these stressors. A number of factors will influence bacterial populations following ICR; these include increased oxygen stress, retrograde flow of colonic contents following removal of the ileocecal valve, inflammatory changes involved in intestinal wound healing, and altered immune function following surgery^19,20^. Bacterial survival mechanisms include the genetic ability to deal with oxygen in the form of aerobic respiration as well as the ability to produce endospores^21^. Here we demonstrate that endospore formation may be a viable strategy for survival in the post-operative gut following intestinal resection. The increased relative fitness of endospores post-operatively may partially explain the predilection for *C. difficile* infection following bowel resection compared to other intra-abdominal surgeries^22^. However, not all spore-forming bacteria carry the pathologic implications of *C. difficile;* recent evidence suggests that over 50% of normal commensal gut microbes are capable of forming spores and a beneficial transfer of spore-forming bacteria can occur between individuals living together^21,23^. Administration of endospores has also been shown to recruit T-regulatory cell populations^24^ although whether Treg cells were involved in maintenance of remission in this study cannot be determined. Importantly, many anaerobic bacteria capable of forming spores (e.g. Clostridiaceae, Lachnospiraceae) also produce short-chain fatty acids, which are beneficial to intestinal health^25^. Interestingly, a lack of *Faecalibacterium prausnitzii* at the time of surgery has been identified to be associated with disease recurrence^6,9^. *F. prausnitzii* are important butyrate-producing bacteria but highly oxygen sensitive^26^ explaining their reduction in patients with active inflammation. A recent study has identified the presence of more than 30 genes related to endospore formation in the genome of *F. prausnitzii*^27^, suggesting that it has the ability to withstand intestinal surgery through endospore formation.

In agreement with previous studies^5–11^, we show that at 6 months following surgery the microbial composition differs between patients in remission and those who have disease recurrence. This is expected as the presence of inflammation alters gut microbial composition^28,29^ and thus a causative relationship cannot be determined. Based on our findings, we propose that two populations within the Firmicutes phylum- those capable of aerobic respiration, and those capable of endospore-formation- possess the capability to endure an operative insult and act as keystone species in the re-colonization of the gut. We speculate that the relative amounts and presence of specific species at the time of surgery may determine how subsequent colonization occurs. Also in agreement with previous studies, we found that the gut microbiota at the time of surgery differed between patients who remained in remission versus those which suffered early disease relapse. De Cruz *et al*.^7^ identified patients who relapsed to have more Streptococcaceae and Enterococcacea and reduced Clostridiales and Bacteroidales at the time of surgery. Further, at the time of surgery, patients who remained in remission had higher amounts of *Faecalibacterium* and *Ruminococcus*, both putative spore-formers. In a similar study, Wright *et al*.^6^ also identified a reduced amount of *F. prausnitzii* and increased levels of *Proteus*, a member of Enterobacteriaceae to be increased. Overall these findings are in agreement with our hypothesis that relative ratios of these functional groups may be the determining factor in gut recolonization and subsequent disease recurrence. Interestingly, recolonization with encapsulated endospores from selected Firmicutes species was recently demonstrated to be effective in preventing recurrence of *Clostridium difficile* infection^30^. The spore preparations used in this study were characterized for spore concentration and absence of residual gram-negative bacteria. The spore communities in these preparations were sequenced using 16S rRNA sequencing and included Clostridiaceae, Erysipelotrichaceae, Eubacteriaceae, Lachnspiraceae, Peptostreptococcaceae and Ruminococcaceae, including members of *Faecalibacterium* genera. Further, by week 8 following treatment, spore-forming bacteria identified in the donor but not in the recipient pre-transplant were shown to have engrafted and to comprise ~30% of the total gut microbial in the recipients. Recipients also showed an expansion of other anaerobic species coupled with a decrease in Enterobacteriaceae following transplant with the isolated spore formulation. These results support our hypothesis that a large number of spore-forming organisms in the human intestine exist and do sporulate and further, have the capacity to modulate gut microbial ecology from a spore form.

Limitations to our study include the fact that our cohort did not receive standardized post-operative care. This led to heterogeneity in terms of anti-inflammatory and antimicrobial regimens, which were underpowered for sub-group analysis. Furthermore, DNA extraction for the purpose of microbial identification is a process that introduces a significant amount of bias. This is especially true given that spore-forming bacteria, when in spore form, may be especially resistant to identification. Finally, PICRUST data regarding the citric acid cycle and sporulation are not direct measure of activity but are an inferred functional capacity. Therefore, caution must be exercised in interpreting these results, as bacterial potential behavior may not reflect actual behavior.

In conclusion, we demonstrate, for the first time, that a dominance of endospore-forming anaerobic bacteria in the ileal mucosa at the time of surgical resection may be associated with maintenance of disease remission, whereas a dominance of aerobic bacteria may be associated with disease recurrence. These findings suggest that a possible strategy to prevent post-operative recurrence of CD is to promote recolonization with encapsulated endospores from selected Firmicutes species. Overall, these findings highlight the importance of overall microbial ecosystem structure in gut health. Gaining an understanding of how microbial communities re-assemble following gut insults has the potential to aid in the development of specific microbial-altering therapy aimed at modulating this process and has profound clinical implications.

## Electronic supplementary material

Supplemental Tables and Figures
