# Supplementary material for: Endospore forming bacteria may be associated with maintenance of surgically-induced remission in Crohn’s disease
Source: Sci Rep. 2018 Jun 27;8:9734. doi: 10.1038/s41598-018-28071-z (PMC6021420; doi:10.1038/s41598-018-28071-z)

Supplementary Information

Endospore forming bacteria may be associated with maintenance of surgically-induced remission in Crohn's disease

Michael R. Laffin[1], Troy Perry[1], Heekuk Park[2], Patrick Gillevet[3], Masoumeh Sikaroodi[3], Gilaad G. Kaplan[4], Richard N. Fedorak[2], Karen Kroeker[2], Levinus A. Dieleman[2], Bryan Dicken[1], Karen L. Madsen[2]

[1]Department of Surgery, University of Alberta, Edmonton, Alberta; [2]Department of Medicine, University of Alberta, Edmonton, Alberta; [3]Microbiome Analysis Center, George Mason University, Manassas, Virginia; [4] Department of Medicine and Community Health Sciences, University of Calgary, Calgary, Alberta

Supplementary Table 1: Rutgeerts score for recurrence of post-operative CD in the neo-terminal ileum

RUTGEERTS SCORE	
**0**	No lesions
**1**	< 5 aphthous lesions
**2**	>5 aphthous lesions with normal mucosa between the lesions, or skip areas of larger lesions or lesions confined to the ilecolonic anastomosis
**3**	Diffuse aphthous ileitis with diffusely inflamed mucosa
**4**	Diffuse inflammation with already large ulcers and/or narrowing

From Rutgeerts *et al.* (1990) [1]

Supplementary Table 2. Patient characteristics.

	Procedure	Rutgeerts score	Indication	Pre-operative biologic	Post-operative biologic	Post-operative Antibiotic	Age at diagnosis	Disease behaviour	Perianal disease	Current Smoker
1	ICR	0	Obstruction	No	No	Ciprofloxacin 14 days	>40	Stricturing	No	No
2	nICR	2	Obstruction	No	Yes	No	17-40	Stricturing	No	No
3	ICR	3	Obstruction	No	No	No	>40	Penetrating	No	No
4	ICR	0	Obstruction	No	Yes	No	17-40	Penetrating	No	No
5	ICR	0	Obstruction	No	No	Metronidazole 14 days	17-40	Stricturing	Yes	No
6	nICR	1	Obstruction	No	No	No	17-40	Stricturing	Yes	Yes
7	ICR	0	Obstruction	No	No	No	17-40	Stricturing	No	No
8	nICR	2	Obstruction	Yes	Yes	No	17-40	Stricturing	No	No
9	nICR	0	Obstruction	No	No	Metronidazole 90 days	<16	Stricturing	Yes	No
10	nICR	2	Obstruction	Yes	Yes	No	17-40	Penetrating	No	No
11	nICR	2	Obstruction	Yes	No	Metronidazole 90 days	17-40	Stricturing	No	No
12	ICR	0	Obstruction	No	No	No	17-40	Stricturing	No	No
13	ICR	1	Obstruction	Yes	Yes	No	17-40	Penetrating	No	No
14	nICR	0	Obstruction	No	No	No	17-40	Stricturing	No	No
15	nICR	2	Obstruction	No	No	No	17-40	Stricturing	No	Yes
16	ICR	0	Obstruction	No	No	Metronidazole 90 days	17-40	Stricturing	No	No
17	ICR	0	Obstruction	Yes	No	Metronidazole 90 days	<16	Penetrating	No	No
18	ICR	1	Obstruction	Yes	Yes	Metronidazole 14 days	<16	Stricturing	No	No
19	ICR	0	Obstruction	Yes	Yes	No	17-40	Stricturing	No	No
20	ICR	0	Obstruction	Yes	Yes	No	17-40	Stricturing	No	No

21	ICR	2	Obstruction	No	No	No	<16	Stricturing	Yes	No
22	ICR	0	Fistula	Yes	Yes	Metronidazole 90 days	17-40	Penetrating	No	No
23	ICR	2	Obstruction	Yes	Yes	No	17-40	Penetrating	No	No
24	ICR	0	Inflammation	No	Yes	No	<16	Inflammatory	No	No
25	ICR	0	Fistula	No	No	Metronidazole 14 days	17-40	Penetrating	No	No
26	nICR	1	Inflammation	No	No	No	17-40	Inflammatory	No	No
27	nICR	2	Obstruction	No	No	No	17-40	Stricturing	No	Yes
28	ICR	1	Fistula	No	Yes	Metronidazole 90 days	17-40	Penetrating	Yes	No
29	ICR	2	Fistula	Yes	Yes	Metronidazole 90 days	17-40	Penetrating	Yes	No
30	ICR	0	Obstruction	No	Yes	No	<16	Stricturing	No	Yes
31	nICR	1	Obstruction	Yes	Yes	No	17-40	Stricturing	No	Yes
32	ICR	0	Inflammation	Yes	Yes	Metronidazole 90 days	17-40	Stricturing	No	No
33	nICR	2	Obstruction	Yes	Yes	Metronidazole 70 days	17-40	Stricturing	No	No
34	ICR	3	Obstruction	Yes	No	No	17-40	Stricturing	No	No
35	ICR	0	Obstruction	Yes	Yes	Metronidazole 90 days	>40	Penetrating	No	No
36	ICR	2	Obstruction	No	No	None	>40	Penetrating	No	No
37	ICR	0	Fistula	Yes	Yes	Metronidazole 30 days	<16	Penetrating	No	No
38	nICR	1	Obstruction	Yes	Yes	No	17-40	Stricturing	No	No
39	nICR	0	Obstruction	No	No	No	>40	Stricturing	No	No
40	nICR	0	Obstruction	Yes	Yes	No	<16	Stricturing	Yes	No
41	nICR	3	Obstruction	Yes	Yes	No	17-40	Penetrating	No	No
42	nICR	0	Obstruction	Yes	Yes	Metronidazole 90 days	17-40	Stricturing	No	No

43	nICR	1	Obstruction	No	No	Ciprofloxacin 28 days	>40	Penetrating	No	No
44	nICR	3	Obstruction	No	No	Ciprofloxacin 28 days	17-40	Stricturing	No	No
45	nICR	0	Fistula	Yes	Yes	No	<16	Penetrating	No	No

Supplementary Table 3: Classification of families within the Firmicutes phylum in terms of their member species ability to perform aerobic respiration or undergo sporogenesis

Class	Order	Family	Species capable of aerobic respiration	Spore-forming member species
**Bacilli**	Bacillales	Bacillales	Yes	Yes
		Listeriaceae	Yes	No
		Paenibacillaceae	Yes	Yes
		Staphylococcaceae	Yes	No
		Thermoactinomycetaceae	Yes	Yes
	Lactobacillales	Aerococcaceae	Yes	No
		Carnobacteriaceae	Yes	No
		Enterococcaceae	Yes	No
		Lactobacillaceae	Yes	No
		Leuconostocaceae	Yes	No
		Streptococcaceae	Yes	No
**Clostridia**	Clostridiales	Clostridiaceae	No	Yes
		Eubacteriaceae	No	No
		Heliobacteriaceae	Yes	Yes
		Lachnospiraceae	No	Yes
		Peptococcaceae	No	Yes
		Peptostreptococcaceae	Yes	Yes
		Ruminococcaceae	No	Yes
	Halanaerobiales	Halanaerobiaceae	No	No
	Thermoanaerobacteriales	Thermodesulfobiaceae	No	No
**Erysipelotrichia**	Erysipelotrichales	Erysiopelotrichaceae	Yes	Yes
**Negativicutes**	Selenomonadales	Acidaminococcaceae	No	Yes
		Veillonellaceae	No	Yes

Only families that were detected during sequencing are included above.

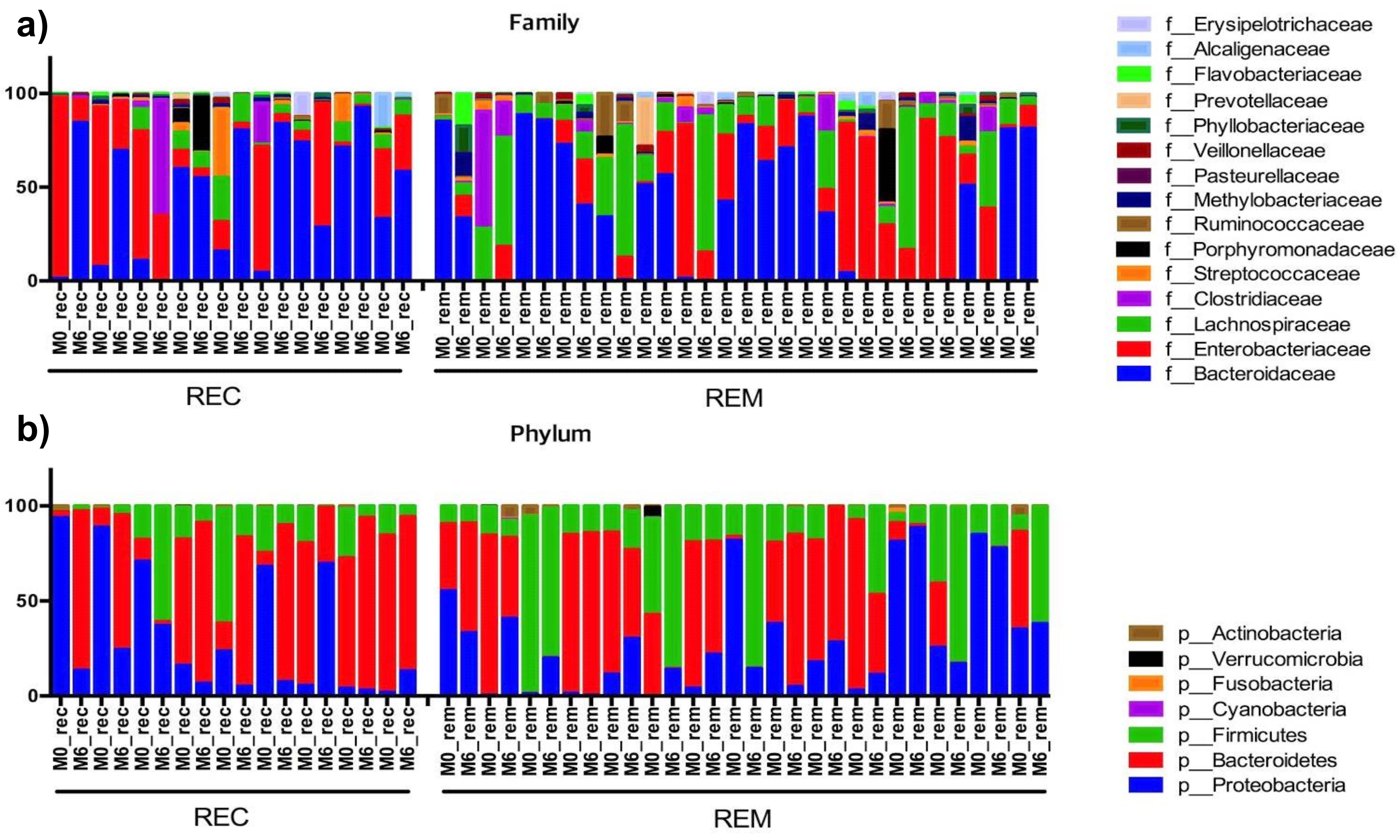
a)
Family
100
50
0
M0_rec
M6_rec
M0_rem
M6_rem
REC
REM
f__Erysipelotrichaceae
f__Alcaligenaceae
f__Flavobacteriaceae
f__Prevotellaceae
f__Phyllobacteriaceae
f__Veillonellaceae
f__Pasteurellaceae
f__Methylobacteriaceae
f__Ruminococcaceae
f__Porphyromonadaceae
f__Streptococcaceae
f__Clostridiaceae
f__Lachnospiraceae
f__Enterobacteriaceae
f__Bacteroidaceae
b)
Phylum
100
50
0
M0_rec
M6_rec
M0_rem
M6_rem
REC
REM
p__Actinobacteria
p__Verrucomicrobia
p__Fusobacteria
p__Cyanobacteria
p__Firmicutes
p__Bacteroidetes
p__Proteobacteria

Supplementary Figure 2. Mucosal cytokine levels in biopsies taken at the time of surgery (A). Levels of cytokines in relation to endospore:aerobic bacteria ratios (B).

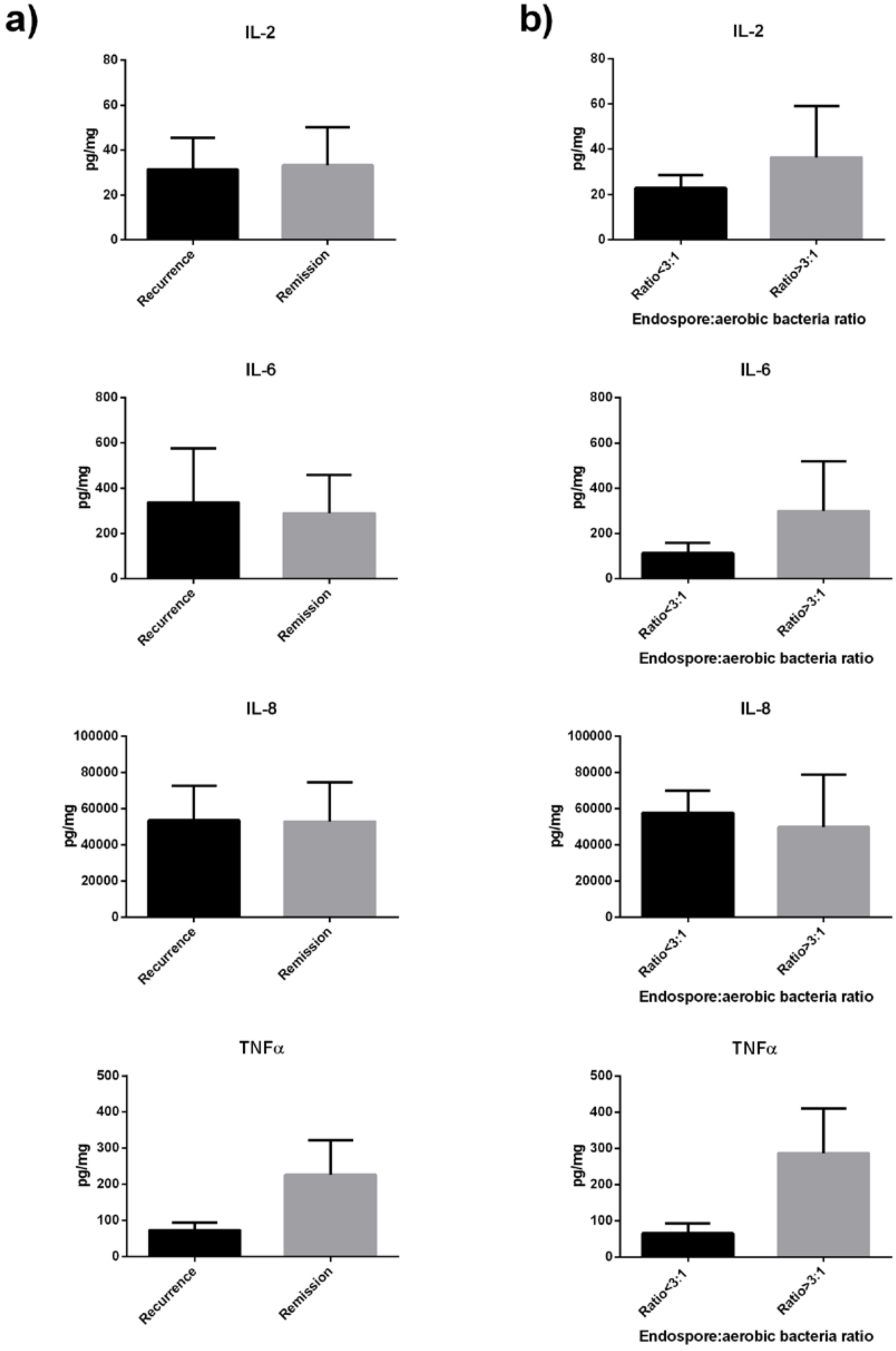

Supplement: Supplementary file 1 — Supplemental Tables and Figures [file 41598_2018_28071_MOESM1_ESM.pdf]
